# Coupling of within-person changes in sleep quality and subjective cognition in community-dwelling adults

**DOI:** 10.1080/13607863.2025.2597964

**Published:** 2026-01-13

**Authors:** Jose A. Diaz, Soomi Lee, Lynn M. Martire, Martin J. Sliwinski

**Affiliations:** Center for Healthy Aging and Department of Human Development and Family Studies, The Pennsylvania State University, University Park, PA, USA

**Keywords:** Within-person change, sleep quality, subjective cognition, coupling

## Abstract

**Objectives:**

Subjective cognitive decline (SCD) is an early indicator of cognitive impairment and dementia risk. Sleep quality, essential for cognitive health, often deteriorates with age. Intraindividual changes in sleep quality may be linked to concurrent changes in subjective cognition; this dynamic ‘coupling effect’ could inform strategies for prevention before objective impairments emerge but remains unexamined longitudinally. We investigated this interplay in older adults over multiple years.

**Method:**

Utilizing the ‘nlme’ R package, we analyzed nine waves of sleep quality and subjective cognition data from the Transitions in Health and Relationships study (*n* = 131, ages 59–94 at baseline, 63% female) in hierarchical multilevel models, with random intercepts and slopes.

**Results:**

Multilevel models indicated changes in sleep quality were positively associated with concurrent changes in subjective cognition, independent of baseline sleep quality, and sociodemographic covariates.

**Conclusion:**

Findings highlight the importance of monitoring within-person sleep quality changes as a potential indicator of SCD in older adults. Further studies may help identify pathways to support cognitive health in aging populations.

## Introduction

Subjective cognitive complaints are increasingly recognized as early indicators of future cognitive impairment and dementia. These self-reported concerns often arise even when objective cognitive test results are normal, suggesting they may serve as an early warning sign of underlying pathology or cognitive decline (Jessen et al., 2014; Pike et al., 2022). As evidence grows that subjective cognitive concerns can precede measurable impairments, identifying the factors that shape these perceptions is vital for recognizing at-risk individuals earlier in the aging process. One key factor that may be associated with subjective cognitive concerns is sleep quality, which was a focus of the current study. First, we describe previous research on subjective cognition, followed by an overview of sleep quality and its associations with cognition.

### Subjective cognition

Subjective cognition refers to an individual’s perception of their cognitive abilities, such as memory, attention, and executive function. Longitudinal studies have shown that older adults with subjective cognitive decline (SCD) have a higher risk of developing dementia compared to those without such complaints (van Harten et al., 2018; Mitchell et al., 2014; Pike et al., 2022; Slot et al., 2019). Furthermore, baseline SCD predicts the rate of objective cognitive decline over time, with higher initial SCD linked to faster decline (Slot et al., 2019; Vogel et al., 2017). Although individual cross-sectional studies have shown mixed results regarding the relationship between SCD and objective cognitive performance (e.g. Amariglio et al., 2011), a meta-analysis and systematic review of cross-sectional studies suggests a weak but consistent association (Burmester et al., 2016). Further, there is evidence that reports of SCD, with normal objective cognition measures, are associated with increased risk of later objective decline and Alzheimer’s Disease and Related Dementias (ADRDs) (Jessen et al., 2014). Importantly, individuals with SCD but no measurable cognitive deficits often exhibit structural and functional brain changes similar to those seen in mild cognitive impairment (MCI) and Alzheimer’s Disease (AD; Haley et al., 2009; Saykin et al., 2006; Si et al., 2020). This suggests that subjective cognitive concerns may reflect subtle brain changes that occur earlier than detectable deficits in objective cognitive tests, consistent with the cognitive and brain reserve theories (Stern, 2012). In this framework, SCD may reflect an individual’s awareness of compensatory brain processes that temporarily preserve cognitive performance, despite underlying pathological changes progress.

These findings indicate that monitoring of subjective cognition could be a valuable tool for early identification of individuals at high risk for future objective decline or development of MCI and ADRDs. This would allow for earlier interventions to prevent or delay onset of objective cognitive decline, MCI, and ADRDs in these high-risk individuals, and could additionally serve as an indicator to closely monitor objective cognition for signs of decline. It is also easier to implement in population-based surveys than lab-based objective cognitive measures. Traditionally, SCD is defined by asking individuals to retrospectively report whether they perceive a decline in their cognitive abilities. Our approach differs in that we assess subjective cognition repeatedly over time, allowing us to directly observe changes rather than relying on a single, retrospective self-assessment. This longitudinal method provides a more precise and dynamic picture of subjective cognition, enabling us to capture not only trajectories of decline but also periods of stability or even improvement.

### Sleep quality and cognition

Sleep quality can be measured subjectively or objectively. Objective assessments, such as polysomnography or actigraphy, quantify difficulties initiating or maintaining sleep including sleep onset latency (SOL), wake after sleep onset (WASO), and sleep efficiency (SE) (O’Donnell et al., 2009). In contrast, subjective sleep quality reflects individuals’ perceptions and satisfaction with their sleep, typically measured using questionnaires like the Pittsburgh Sleep Quality Index (PSQI) or sleep diaries (Bin, 2016; Buysse et al., 1989; Casagrande et al., 2022; Nelson et al., 2022; O’Donnell et al., 2009). Sleep quality—whether measured objectively or subjectively—is critical for maintaining cognitive health, with restorative processes offering protection against cognitive decline, including AD/ADRD (Bartolo & Hill, 2022; Chong et al., 2022; Scott et al., 2021). Sleep quality is a well-established factor in brain health, with poor sleep quality linked to an increased risk of AD/ADRD, cognitive decline, cardiovascular disease, and poorer objective cognitive performance in older adults, while both objectively-measured and perceived good sleep quality are linked to better cognitive performance in older adults (Besedovsky et al., 2019; Crowley, 2011; Espiritu, 2008; Mander et al., 2017; Miller et al., 2014; Ohayon et al., 2004; Pace-Schott & Spencer, 2011; Qin et al., 2023). Notably, a study that simultaneously examined multiple objective and subjective sleep characteristics shows that subjective sleep quality is most strongly associated with adult well-being measured by perceived stress and chronic physical conditions (Lee & Lawson, 2021). In this study, we focus on subjective sleep quality to examine whether changes in perceived sleep quality correspond to changes in subjective cognition.

Despite the importance of subjective cognition as an early marker of cognitive decline, limited research has examined how certain lifestyle factors, such as sleep quality, impact subjective cognition. There are many lifestyle factors that may be associated with subjective cognition in older adults, but sleep quality is of particular note as it may independently impact these lifestyle factors, alongside its own association with subjective cognition. One example of this is physical activity, as prior research has found that engaging in physical activity is associated with a lower risk of SCD; however, sleep quality has been found to have a significant association with physical activity, with better sleep quality predicting higher levels of physical activity in older adults (Holfeld & Ruthig, 2014; Xu et al., 2023). Thus, sleep quality may influence subjective cognition both directly and indirectly by facilitating lifestyle behaviors such as physical activity that support cognitive health, further highlighting the importance of studying the relationship between SQ and cognition.

Studies on the relationship between subjective sleep quality and current objective cognition specifically, have found mixed results. Studies have found a small significant relationship with global cognition, and one review reported a moderate positive relationship; however, several studies found that subjective sleep quality was only associated with a few subsets of cognitive domains, not global cognition (Bastien et al., 2003; Campbell et al., 2022; Casagrande et al., 2022; Miyata et al., 2013; Nebes et al., 2009; Saint Martin et al., 2012; Waller et al., 2016; Zavecz et al., 2020). Additionally, in longitudinal studies, poor subjective sleep quality has been found to be associated with objective cognitive decline and later development of MCI and ADRD (Ancoli-Israel et al., 2022; Chen et al., 2022; Chen and Wang, 2022; Jelicic et al., 2002; Li et al., 2022; Potvin et al., 2012; Tsapanou et al., 2015).

In contrast to research on sleep quality and its associations with objective cognition and decline, the few studies on the relationship between subjective sleep quality and subjective cognition provide evidence of a stronger potential link. For example, a large cross-sectional study in Korea found that individuals with poor subjective sleep quality had significantly higher odds of reporting SCD, independent of objective cognitive measures (Kim et al., 2021). Similarly, other studies indicated that poor sleep quality and sleep disturbances were associated with increased reports of subjective cognitive difficulties and worse subjective cognition, suggesting that these perceptions may be interconnected (Lee et al., 2020; Nicolazzo et al., 2021; Tsapanou et al., 2019). However, findings have not been entirely consistent across studies. For instance, one study initially found a significant positive correlation between the PSQI and a measure of subjective cognitive functioning, but after adjustment for confounding variables (e.g. anxiety, depression, etc.), this relationship was no longer significant (Saint Martin et al., 2012). Further, few longitudinal studies have investigated how changes in sleep quality may be linked to changes in subjective cognition over time. One notable exception is a study of women with breast cancer, which found that declines in sleep quality predicted concurrent declines in subjective cognition (Ancoli-Israel et al., 2022). The limited and mixed evidence from existing studies underscores the need for longitudinal research to better understand the temporal relationship between subjective sleep quality and subjective cognition.

Given the potential for subjective cognition to serve as an early indicator of cognitive decline, understanding how fluctuations in sleep quality are coupled with these self-perceptions is crucial. Investigating the dynamic relationship between sleep and subjective cognition over time could yield valuable insights, helping to identify opportunities to promote healthier aging and address cognitive decline before objective impairments emerge. However, there are additional variables that may underlay or have a mediating effect on the relationship between subjective sleep quality and cognition. Anxiety and depression have both been found to be associated with SCD and there is evidence that they may mediate the relationship between subjective sleep quality and SCD, as such, controlling for these variables is necessary to accurately model and isolate the unique association between sleep quality and subjective cognition (Sun et al., 2024).

### Age-related changes to sleep

Individuals experience changes to their sleep architecture as they age, with age-related changes seen as early as middle adulthood. Time spent in different sleep stages changes in normal aging, with older adults spending less time in slow wave sleep and rapid eye movement (REM) sleep with increasing age, instead increasingly spending their time asleep in stage 1 and 2 non-REM sleep (Cooke and Ancoli-Israel, 2011; Espiritu, 2008; Mander et al., 2017; Miller et al., 2014; Ohayon et al., 2004; Pace-Schott & Spencer, 2011). Older adults also commonly experience changes to other aspects of objective sleep quality, such as increased SOL, lower SE, more WASO, and lower total sleep duration, which may interrupt the time they spend in sleep stages associated with objective cognition (Cooke and Ancoli-Israel 2011; Miller et al., 2014; Ohayon et al., 2004; Yaffe et al., 2014). Further, these aspects of sleep are associated with subjective sleep quality, and the changes seen may reduce ratings of sleep quality as the individual ages, impacting subjective cognition. However, it should be noted that even though sleep quality declines with age, there is significant variability in sleep quality between- and within-people (Gao and Scullin, 2023; Scullin and Gao, 2018). As such, determining how age impacts subjective cognition in older adults is important and age must be controlled in order to determine if subjective sleep quality is independently associated with SCD.

### Current study

The current study examined the coupling relationship between sleep quality and subjective cognition over the course of aging. Coupled change refers to whether variation in one variable, such as sleep quality, is accompanied by concurrent changes in another, such as subjective cognition, within the same person over time (Neubauer et al., 2020; Rush et al., 2019; Sliwinski et al., 2003, 2009). This approach addresses the question: when a person’s sleep quality improves or declines, does their subjective cognition improve or decline simultaneously? Unlike the between-person analyses of correlated change—which examine whether individuals with greater overall changes in one variable also exhibit greater changes in another overall—coupled change focuses on dynamic, within-person interactions across the study period. We estimated the coupling effect by calculating within-person changes from a baseline measure of sleep quality, tracking these changes across multiple time points, and linking the sleep quality changes to simultaneous changes in subjective cognition. We hypothesized that changes in sleep quality over time would be coupled with concurrent changes in subjective cognition, such that improvements in sleep quality would correspond to improvements in subjective cognition. Given that both sleep quality and subjective cognition were self-reported, we tested this hypothesis after controlling for a range of covariates commonly associated with changes in subjective cognition, including baseline age, gender, household income, anxiety, depressive symptoms, and baseline levels of subjective cognition and sleep quality (Hülür et al., 2014; Nebes et al., 2009; Nicolazzo et al., 2021; Saint Martin et al., 2012; Tsapanou et al., 2019; Waller et al., 2016; Zlatar et al., 2018).

## Methods

### Participants

Participants were older adults residing in independent living or retirement communities who took part in the Transitions in Health and Relationships study, a longitudinal observational study examining changes in health and relationships among older adults (Marini et al., 2021; L. Martire et al., 2019). This study was approved by the Pennsylvania State University Institutional Review Board (IRB # CR00024180). Eligibility criteria included the absence of language, hearing, or vision problems that would hinder participation in interviews or surveys, and no significant cognitive impairment, as indicated by a score of 5 or greater on the Memory Impairment Screen (Buschke et al., 1999). Data collection began in 2016 and included two sessions per year, spaced 6 months apart. Each session involved participants completing several questionnaires on demographics (baseline only), physical and mental health, relationships, and behavior.

The current study focuses on a subset of these data, specifically from 10 data collection waves. However, due to complications from the COVID-19 pandemic, our variables of interest were only collected in nine of these waves. At baseline, participants completed an in-person interview, followed by an online Qualtrics survey six months later. This pattern of alternating in-person and online sessions continued for subsequent data collection waves. On online data collection waves, participants received personalized links by email and were provided with telephone assistance if they encountered any technical difficulties. These procedures ensured consistency and data quality across assessment modes. All eligible participants (*n* = 131 at baseline) were included in this analysis, though only 105 completed the final data collection wave due to attrition.

The mean age of participants at baseline was 77.09 years (SD = 9.03), ranging from 58 to 94 years. Approximately 63% of participants were female. The sample lacked racial and ethnic diversity, with 99.23% identifying as non-Hispanic White, and only one individual identifying as Hispanic. Participants were generally well-educated, with 97.71% having completed at least some college education. Additional demographic information is provided in Table 1.

### Measures

*Subjective sleep quality* was measured at each wave using a previously validated scale, the 4-item PROMIS-29 (v2.1) Sleep Disturbance scale (Working Group on Health Outcomes for Older Persons with Multiple Chronic Conditions, 2012). This scale consists of four items (‘How was your sleep quality’; ‘Your sleep was refreshing’; ‘You had a problem with your sleep’; ‘You had difficulty falling asleep’) in which participants were asked to rate their sleep in the past 7 days on a 5-point scale (0–4; Very Poor/Not at all, Poor/A little bit, Fair/Somewhat, Good/Quite a bit, Very Good/Very much). Items three and four were then reverse-coded and used alongside items one and two to calculate a mean sleep quality score for each participant at each wave. In our sample, we found high between-person reliability (0.92), calculated using McDonald’s omega (*ω*).

*Subjective cognition* at each wave was acquired using the 8-item Neuro-QoL v2.0 Cognitive Function Short Form (Cella et al., 2011). The 8-item measure asks participants to indicate how often they have experienced some deficit in cognitive function over the past 7 days (e.g. ‘You had to read something several times to understand it’, ‘Your thinking was slow’, etc.), and how much difficulty they have with cognitively demanding tasks (e.g. ‘Reading and following complex instructions’, ‘Planning for and keeping appointments that are not part of your weekly routine’, etc.). Participants responded on a 5-point scale, with the first four items reverse coded, and a subjective cognition score was calculated by taking the mean of the 8 items. The Neuro-QoL v2.0 Cognitive Function Short Form has previously been found to be reliable, with good internal consistency (Cronbach’s *α*) across all subgroups in a study sample (0.87 to 0.94; Iverson et al., 2021). In the current study, McDonald’s *ω* indicated that there was good between-person reliability (0.95).

#### Covariates.

Several additional variables were included as covariates in the analysis. Time-invariant covariates included baseline participant age, gender, and household income. Race/ethnicity and education were not included as covariates due to the sample being predominantly non-Hispanic White and highly educated. Anxiety and depressive symptoms were included as time-varying covariates and were measured at each wave using the PROMIS-29 Short Form Anxiety and Depression scales (Working Group on Health Outcomes for Older Persons with Multiple Chronic Conditions, 2012).

### Statistical analyses

Analyses were conducted using R statistical software (v4.4.1; R Core Team, 2024). We built several hierarchical multilevel models with random intercepts and slopes using the ‘lme()’ function from the ‘nlme’ R package (3.1.163; J. Pinheiro et al., 2023; J. C. Pinheiro & Bates, 2000). This approach allowed us to model change in subjective cognition over time, and their dynamic within-person relationship with changes in subjective sleep quality, adjusting for baseline values of these main variables and several other covariates.

During data preparation, we recoded the Wave variable so that Wave 1 (baseline) was set to 0 for easier interpretation. We also created a dummy variable, ‘Assessment Mode’, to differentiate between data collected through in-person interviews and online follow-up surveys. Additionally, given that both subjective sleep quality and subjective cognition were self-reported measures, we tested for common method variance (CMV) using confirmatory factor analysis (CFA). Using the ‘lavaan’ R package, we compared a two-factor model (subjective sleep quality and subjective cognition as distinct constructs) against a single-factor model (all items loading on one common method factor) using baseline data (Rosseel, 2012; Rosseel et al., 2025). To test our hypotheses, we developed three hierarchical multilevel models. Model 1 included time (e.g. Wave) and assessment mode (In-person Interview vs. Online Survey) as predictors. This model also included random intercepts and slopes that allowed for variability in both the initial scores (intercept) and the rate of change over waves of the study (slope) across individual participants. In Model 2, additional sociodemographic variables and covariates were introduced, including baseline age, gender, household income, and anxiety and depressive symptom scores.

In Model 3, we accounted for both baseline sleep quality and within-person changes in sleep over time. Baseline sleep quality was grand-mean centered across participants to examine its association with trajectories of subjective cognition. To capture within-person fluctuations in sleep quality, we calculated a change score at each follow-up wave by subtracting an individual’s baseline sleep quality from their sleep quality at that wave (De Looze et al., 2022). This variable was included as both a fixed and random effect to assess whether changes in sleep quality were linked to concurrent changes in subjective cognition.

We also examined whether the relationship between sleep quality and subjective cognition varied based on study wave and assessment mode, allowing us to determine if changes in cognition differed depending on initial sleep quality and whether assessment mode influenced this relationship. Data is available on request. All code used for data cleaning and analysis, including full model specification, is available in the following OSF repository: https://osf.io/gjdev/.

## Results

### Descriptive statistics

Table 2 presents descriptive statistics at baseline for variables of interest. Mean subjective sleep quality at baseline was 2.81 (SD = ±0.82; 0.33–4.00), while mean subjective cognition was 4.20 (SD = ±0.47; 3.00–5.00). To assess whether sleep quality changed significantly over time, we first fit a preliminary longitudinal model. This analysis indicated no significant change in subjective sleep quality across study waves (*β* = −0.01, 95% CI [−0.02, 0.01], *p* = .22). Additionally, the results of the CFA testing for CMV indicated low CMV. The two-factor model fit significantly better than the single-factor model (Δ*χ*^2^ = 192.27, Δ*df* = 1, *p* < .001), and the correlation between subjective sleep quality and cognition factors was low (*r* = 0.257), suggesting that shared method variance is negligible.

### Demographic and covariate effects on subjective cognition

Fixed effect results for Model 1, with wave in study and assessment mode as predictors for subjective cognition, indicated that there was a small but significant decline in subjective cognition across waves (*β* = −0.013, 95% CI [−0.021, −0.005], *p* = .002). Additionally, the fixed effect for assessment mode was significant (*β* = 0.060, 95% CI [0.024, 0.095], *p* = .001). This suggested that on waves where participants completed data collection through an online survey, they reported higher subjective cognition scores, compared to waves where they completed an in-person interview. The random effects results for Model 1 were indicative of the variability in subjective cognition around the intercept (γ_00) across participants and the rate of change over waves of the study (γ_10). Additionally, there was a positive correlation between the random intercept and random slope (*r* = 0.306), which suggests that higher subjective cognition at baseline (intercept) was associated with less decline (or improvements) across waves, or vice-versa, lower subjective cognition at baseline was associated with steeper decline. The results of Model 1, including fixed and random effects, are summarized in Table 3.

In Model 2, demographic and covariate variables were added. Fixed effects results showed that study wave remained significantly negatively associated with subjective cognition (*β* = −0.010, 95% CI [−0.018, −0.001], *p* = 0.025), though the effect was slightly weaker than in Model 1. In contrast, assessment mode remained significantly positively associated with subjective cognition (*β* = 0.075, 95% CI [0.038, 0.113], *p* < .001) but had a stronger effect in Model 2.

Additionally, several of the variables added to this model were significantly associated with subjective cognition. Although the effect was small, baseline age was a significant predictor (*β* = −0.013, 95% CI [−0.021, −0.005], *p* = 0.001), with older age at baseline linked to lower subjective cognition. Household income at baseline showed a significant positive association with subjective cognition (*β* = 0.085, 95% CI [0.022, 0.148], *p* = .009). Both anxiety and depression symptom scores were significantly negatively associated with subjective cognition (*β* = −0.143, 95% CI [−0.201, −0.086], *p* < .001; *β* = −0.114, 95% CI [−0.186, −0.042], *p* = .002), indicating that higher anxiety or depression scores were linked to lower subjective cognition. Gender was not a significant predictor (*p* = .076), suggesting no significant gender differences in subjective cognition.

The random effects results were similar to those in Model 1, with a slight reduction in random variation around the intercept and the rate of change over waves. Additionally, the correlation between the random intercept and slope was lower than in Model 1 (*r* = 0.163). Despite the inclusion of significant covariates, the primary fixed effects remained largely unchanged from Model 1. A summary of the fixed and random effects results for Model 2 is presented in Table 4.

### Effects of baseline and change in sleep quality on subjective cognition

Model 3 expanded on previous models by incorporating both baseline sleep quality and within-person changes in sleep quality as predictors, along with their interactions with study wave and assessment mode. Additionally, change in sleep quality was included as a random effect. The results of Model 3, both unadjusted and fully adjusted for covariates, are summarized in Table 5.

In the unadjusted Model 3, the fixed effect of baseline sleep quality was significantly positively associated with subjective cognition (*β* = 0.104, 95% CI [0.017, 0.191], *p* = .020), indicating that better sleep quality at baseline was associated with better subjective cognitive function at baseline. Similarly, within-person changes in sleep quality from baseline were significantly positively associated with subjective cognition (*β* = 0.099, 95% CI [0.059, 0.139], *p* < .001), meaning that an increase in sleep quality at a given wave corresponded with an increase in subjective cognition at that wave.

In the fully adjusted model, baseline sleep quality was no longer a significant predictor (*p* = 0.167), while within-person changes in sleep quality from baseline remained significantly positively associated with subjective cognition (*β* = 0.062, 95% CI [0.020, 0.103], *p* = .004), indicating that improvements in sleep quality at a given wave were linked to concurrent increases in subjective cognition, even after adjusting for baseline sleep quality. This finding supports the presence of a coupling effect between changes in sleep quality and changes in subjective cognition. In this model, age at baseline (*p* < .001), household income at baseline (*p* = .004), anxiety scores (*p* < .001), and depression scores (*p* = 0.005) all remained significant. Importantly, interaction effects—such as those between study wave and baseline sleep quality or assessment mode and baseline sleep quality—were not significant (*ps* = .139 and .965 respectively). The random effects results showed that the variance of the intercept was 0.1047, while the variance for the slopes of study wave and change in sleep quality were 0.0004 and 0.0074, respectively. As in previous models, the correlation between the random intercept and the slope of study wave remained positive (*r* = 0.116). The slope of change in sleep quality showed a moderate inverse correlation with the random intercept variance (*r* = −0.308) and a moderate positive correlation with study wave (*r* = 0.317).

## Discussion

In this study, we examined how longitudinal changes in subjective sleep quality were associated with changes in subjective cognition in a sample of older adults. We found that changes in sleep quality from baseline at a wave were coupled with changes in subjective cognition in the same direction, even when adjusting for baseline subjective sleep quality. This was in line with our hypothesis that there would be a positive association between changes at a wave.

Baseline subjective sleep quality was significantly associated with subjective cognition in the unadjusted Model 3 but did not remain significant in the fully adjusted model. Prior research has found significant cross-sectional associations between sleep quality and cognition in older adults, with poor sleep quality increasing the risk of cognitive decline (Andreasson et al., 2021; Kim et al., 2021; Leng et al., 2020; Nicolazzo et al., 2021; Simoes Maria et al., 2020; Tsapanou et al., 2019). One potential explanation for the null cross-sectional association in our study is that fully adjusted models controlled for depression and anxiety, which may be strong risk factors for both poor sleep quality and subjective cognition.

Although there was no evidence of average sleep quality decline across study waves, within-person increases in sleep quality at a given wave were associated with concurrent improvements in subjective cognition. This suggests that sleep quality varies significantly within individuals over time, a pattern not captured by average trends across all participants. More importantly, these individual fluctuations in sleep quality appear to play a role in how individuals perceive their own cognitive function, even when accounting for baseline sleep quality.

Additionally, the effect of changes in sleep quality on subjective cognition (*β* = 0.062, 95% CI [0.020, 0.103], *p* = .004) was larger than that of the individuals baseline age (*β* = −0.01, 95% CI [−0.02, −0.01], *p* = .001). These results suggest that monitoring sleep quality at an individual level could serve as an early detection tool for SCD, particularly when sleep quality declines over time, regardless of baseline levels. Moreover, since sleep quality is a modifiable risk factor, interventions aimed at preventing SCD—and ultimately objective cognitive decline—could target sleep improvement as a potential strategy. Evidence for the impact of close relationships on sleep quality in later adulthood (Marini et al., 2023; L. M. Martire et al., 2013) suggests that such interventions might usefully target interpersonal stressors.

Our study found that while subjective cognition declined over time in older adults, subjective sleep quality remained stable across study waves. Although the decline in subjective cognition was modest, it aligns with our hypothesis and expectations—gradual changes over time rather than sharp declines over short periods. Additionally, the stability of subjective sleep quality is consistent with previous research showing that sleep patterns, including subjective sleep quality, remain relatively stable over a 10-year period in aging adults (Lee et al., 2024).

The results of these models also suggest that there is a differential effect for higher baseline levels of subjective cognition on their rate of change across waves. Specifically, higher levels of subjective cognition at baseline were associated with less decline (or improvements) in subjective cognition across waves. However, having lower subjective cognition at baseline was associated with greater improvements in subjective cognition in response to an increase in sleep quality, compared to those with higher subjective cognition at baseline. In other words, this finding indicates that the strength of the coupling effect between change in sleep quality and subjective cognition over time is greater for individuals who started out with more negative views of their cognition.

### Limitations

While this study has several strengths, including the use of longitudinal data with nine waves and finding that a decrease in sleep quality is a novel risk factor for SCD, it has limitations. Our study relied on self-report measures of sleep quality and subjective cognition, with these instruments capturing individuals’ perceived experiences of sleep and cognitive function—an important and conceptually distinct aspect of brain health that complements, rather than replaces, objective indicators. The absence of concurrent objective measures (e.g. actigraphy or performance-based cognitive tasks) limits our ability to directly compare subjective and objective indices or to identify the extent to which they converge or diverge over time. Likewise, the absence of objective measures limits our ability to test for or correct common method bias, though a test of CMV for sleep quality and subjective cognition indicated low CMV. Our longitudinal, within-person analytical approach that focuses on change over time, rather than cross-sectional association, as well as, the inclusion of anxiety and depression as covariates to account for shared affective influences on self-reports, may in part mitigate this concern. Although our model controlled for baseline participant age, our small sample size and age range limited our ability to conduct an age-stratified analysis to identify potential age-related heterogeneity in our results. Future studies should recruit a larger sample of adults from mid-life to older adulthood to examine potential age moderation. The length of time between waves (6 months) allowed us to determine how changes in sleep quality were associated with changes in subjective cognition over approximately 5 years of aging. However, future research should combine subjective and objective measures within a longitudinal or measurement burst (Stawski et al., 2015) framework to better capture daily fluctuations in sleep and cognition. Such multi-method approaches would provide a more comprehensive understanding of how subjective experiences and physiological processes co-vary and contribute to cognitive health in aging. Additionally, the study’s findings may have limited generalizability due to the lack of variation in key demographic factors such as race/ethnicity, socioeconomic status, and education. Future research should include more diverse populations, including individuals from less privileged backgrounds, to strengthen scientific inference and enhance the generalizability of findings. Further studies should also explore strategies to improve subjective sleep quality in older adults as a potential intervention to prevent or delay SCD.

### Conclusions

This study advances our understanding of the dynamic relationship between sleep quality and subjective cognition by demonstrating that within-person fluctuations in sleep quality are meaningfully linked to concurrent changes in cognitive self-perception over time. Unlike prior research that has largely focused on between-person differences or static associations, our findings highlight the importance of tracking sleep-cognition coupling within individuals. This novel approach suggests that monitoring sleep quality changes may offer a valuable, early indicator of SCD. Given that sleep quality is a modifiable factor, future interventions targeting sleep improvements could play a critical role in preserving cognitive health in aging populations.

## Figures and Tables

**Table 1.** Participant demographics at baseline.

Variable	*N* = 131^a^

Age (years)	77.09 (±9.03; 58.00–94.00)
Female	83 (63.36%)
Race	
American Indian or Alaska Native	0 (0.00%)
Asian	1 (0.77%)
Black	0 (0.00%)
Native Hawaiian	0 (0.00%)
White	129 (99.23%)
Other	0 (0.00%)
Hispanic	1 (0.78%)
Education	
HS/GED or below	3 (2.29%)
At least some college or above	128 (97.71%)
Household income	
<$5000	0 (0.00%)
$5000-$15,000	0 (0.00%)
$15,001-$30,000	2 (1.82%)
$30,001-$50,000	13 (11.82%)
$50,001-$75,000	32 (29.09%)
$75,001-$100,000	29 (26.36%)
>$100,000	34 (30.91%)

a Mean (±SD; range); *n* (%).

**Table 2.** Descriptive statistics at baseline.

Variable	*N* = 131^a^

Subjective sleep quality	2.81 (±0.82; 0.33–4.00)
Subjective cognitive function	4.20 (±0.47; 3.00–5.00)
Depressive symptoms score	0.27 (±0.48; 0.00–2.50)
Anxiety score	0.34 (±0.51; 0.00–2.75)

a Mean (±SD; range).

**Table 3.** Model 1—study wave and assessment mode.

Coeffcient	Subjective cognition			
	Estimates	Standard error	Confidence interval (95%)	*p*

Intercept	4.26	0.04	4.19–4.33	<.001
Wave	−0.01	0.00	−0.02–−0.00	.002
Assessment mode: survey	0.06	0.02	0.02–0.10	.001
**Random effects**				
*σ* ^2^	0.07			
*τ*_00 PID_	0.14			
*τ*_11 PID.Wave_	0.00			
*ρ*_01 PID_	0.31			
ICC	0.71			
*N*_PID_	131			
Observations	1053			
Marginal *R*^2^/conditional *R*^2^	0.005/0.707			

**Table 4.** Model 2—demographic and covariate predictors of subjective cognition.

Coeffcient	Subjective cognition			
	Estimates	Standard error	Confidence interval (95%)	*p* ^a^

Intercept	4.77	0.38	4.02–5.52	**<.001**
Wave	−0.01	0.00	−0.02–−0.00	**.025**
Assessment mode: survey	0.08	0.02	0.04–0.11	**<.001**
Age at baseline	−0.01	0.00	−0.02–−0.01	**.001**
Gender: female	0.13	0.07	−0.01–0.27	.076
Household income at baseline	0.08	0.03	0.02–0.15	**.009**
Anxiety score	−0.14	0.03	−0.20–−0.09	**<.001**
Depression score	−0.11	0.04	−0.19–−0.04	**.002**
**Random effects**				
σ^2^	0.07			
τ_00 PID_	0.10			
τ_11 PID.Wave_	0.00			
ρ_01 PID_	0.16			
ICC	0.64			
*N*_PID_	110			
Observations	881			
Marginal *R*^2^/conditional *R*^2^	0.186/0.711			

a Bold values indicate significant *p*-values.

**Table 5.** Model 3—effects of baseline and change in sleep quality on subjective cognition.

Coefficient	Unadjusted				Adjusted			
	Estimates	Standard error	Confidence interval (95%)	*p*	Estimates	Standard error	Confidence interval (95%)	*p*

Intercept	4.25	0.04	4.18–4.32	**<.001**	4.75	0.39	4.00–5.50	**<.001**
Wave	−0.01	0.00	−0.02–−0.00	**.002**	−0.01	0.00	−0.02–−0.00	**.017**
Sleep quality at baseline	0.10	0.04	0.02–0.19	**.020**	0.06	0.04	−0.03–0.15	.167
Assessment mode: survey	0.07	0.02	0.03–0.10	**<.001**	0.08	0.02	0.04–0.12	**<.001**
Change in sleep quality from baseline	0.10	0.02	0.06–0.14	**<.001**	0.06	0.02	0.02–0.10	**.004**
Wave × sleep quality at baseline	0.01	0.01	−0.00–0.02	.144	0.01	0.01	−0.00–0.02	.139
Sleep quality at baseline × Assessment mode: survey	−0.00	0.02	−0.05–0.04	.972	0.00	0.02	−0.05–0.05	.965
Age at baseline					−0.01	0.00	−0.02–−0.01	**.001**
Gender: female					0.14	0.07	−0.00–0.28	.055
Household income at baseline					0.09	0.03	0.03–0.16	**.004**
Anxiety score					−0.13	0.03	−0.19–−0.07	**<.001**
Depression score					−0.10	0.04	−0.18–−0.03	**.005**
**Random effects**								
*σ* ^2^	0.07				0.07			
*τ*_00_	0.14 _PID_				0.10 _PID_			
*τ*_11_	0.00 _PID.Wave_				0.00 _PID.Wave_			
	0.01 _PID.sleepChange_				0.01 _PID.sleepChange_			
*ρ*_01_	0.24				0.12			
	−0.08				−0.31			
ICC	0.71				0.65			
*N*	131_PID_							
Observations	1053				881			
Marginal *R*^2^/conditional *R*^2^	0.041/0.726				0.223/0.727			

Bold values indicate significant *p*-values.
